# Moe culture and transcultural resonance: a cross-cultural case study of a cute IP on Douyin and TikTok

**DOI:** 10.3389/fpsyg.2026.1925265

**Published:** 2026-09-14

**Authors:** Jindan Hou, Hanyi Zheng, Shaoshuo Cai

**Affiliations:** 1 School of Journalism, Yunnan University, Kunming, China; 2 School of Journalism and Communication, Henan University, Zhengzhou, China; 3 School of Culture and Media, Hubei Preschool Teachers College, Wuhan, China

**Keywords:** digital tribes, generation z, moe culture, symbolic consumption, transcultural resonance

## Abstract

The global circulation of cute intellectual properties (IPs) has emerged as a distinctive affective-cultural phenomenon, yet the psychological mechanisms through which such IPs generate transcultural resonance remain undertheorized. This study investigates how moe aesthetics facilitate transcultural engagement through symbolic consumption and digital tribal practices, using the Muay Thai DunDun Chicken IP on Douyin and TikTok as a cross-cultural case study. Drawing on 446 high-engagement videos and approximately 332,000 characters of user comments, the study employed sentiment analysis, thematic coding, and cross-cultural comparative analysis to examine affective patterns and participatory practices across the two platforms. The findings reveal that moe aesthetics function as a broadly shared affective trigger that elicits instinctive emotional responses across cultural boundaries, suggesting a common perceptual basis while acknowledging culturally situated interpretive frameworks. However, the interpretive frameworks through which users make sense of their affective experience are culturally situated: Chinese users emphasize product attributes and functional value, reflecting a low-context cultural orientation, while Thai users emphasize cultural identity and symbolic meaning, reflecting a high-context cultural orientation. Thematic coding identified nine participatory practices organized into a three-layer model of transcultural resonance: Sensory Drive, Symbolic Production, and Tribal Autonomy. These layers reveal a conceptual framework through which cute IPs generate shared meaning, from broadly shared affective appeal through participatory co-creation to self-organized community governance that sustains resonance over time. The study extends transcultural resonance theory to the domain of cute IP engagement and refines the concept of moe power by demonstrating that affective appeal is not uniform across contexts but is actively shaped by local interpretive frameworks and participatory practices. These findings contribute to cultural psychology by specifying how biological universals and cultural particulars interact in digitally mediated contexts to produce shared affective experiences that transcend cultural boundaries.

## 1 Introduction

Over the past two decades, a distinctive affective-cultural phenomenon originating in Japanese otaku subcultures has gradually permeated global consumer cultures, entertainment industries, and digital social practices. This phenomenon, known as moe, refers to the intense emotional attachment and affectionate response that audiences develop toward fictional characters, often triggered by specific visual and personality cues such as large eyes, childlike facial features, vulnerability, or endearing idiosyncrasies (Galbraith, 2014). Unlike the broader concept of cuteness (kawaii), which primarily denotes an attribute of a stimulus – typically infantile physical features that evoke caregiving responses (Lorenz, 1943) – moe emphasizes an active, relational affective bond between the perceiver and the perceived character or object (Galbraith, 2014). This distinction carries significant psychological implications: cuteness is passively perceived, whereas moe is actively constructed through emotional investment, imaginative engagement, and sustained para-social interaction (Galbraith, 2014).

The psychological mechanisms underlying moe perception have attracted increasing scholarly attention across disciplines. At the most fundamental level, the baby schema – a set of infantile physical features including a large head, round face, big eyes, and chubby cheeks – has been shown to reliably trigger caregiving instincts and positive affect across cultures (Lorenz, 1943; Nittono et al., 2021). Neuroimaging studies have further demonstrated that exposure to baby schema features activates the nucleus accumbens and orbitofrontal cortex, brain regions associated with reward processing and positive emotional experience (Kringelbach et al., 2008; Glocker et al., 2009). These neural responses appear to be cross-culturally consistent, suggesting a biological substrate for cuteness perception that may operate similarly across cultures (Nittono et al., 2021). However, recent research has also revealed important cultural variations in how cuteness is conceptualized, expressed, and valued across different societies. Nittono et al. conducted a large-scale cross-cultural survey across Japan, the United States, and Israel, finding that while conceptions of cuteness showed remarkable cross-cultural resemblance, significant differences emerged in attitudes toward cuteness and its social functions. These findings underscore the need to examine moe and cuteness not merely as universal biological responses but as culturally situated psychological phenomena whose meanings and functions are actively shaped by local cultural contexts.

Beyond its biological and perceptual foundations, moe culture raises important questions about emotional attachment, para-social relationships, and identity formation in digitally mediated environments. Galbraith argued that moe represents a form of active emotional projection in which fans invest significant affective energy in fictional characters, often developing para-social bonds that rival real-world relationships in their emotional intensity and personal significance (Galbraith, 2014). This observation aligns with broader research on para-social relationships, which has documented how media audiences form one-sided emotional connections with media figures, experiencing feelings of friendship, trust, and intimacy despite the absence of reciprocal interaction (Horton and Wohl, 1956; Tukachinsky, 2011). In contemporary digital contexts, these para-social dynamics have been amplified by social media platforms, which enable fans to share their emotional investments, co-create content, and form collective identities around shared affective attachments (Jenkins, 2006).

The rise of moe culture has also generated significant commercial and cultural momentum beyond Japan, particularly in East and Southeast Asian markets. China has emerged as a major site of moe culture production and consumption, with domestic cute intellectual properties (IPs) achieving substantial commercial success and, in some cases, transcultural reach (Zhao and Niu, 2025). These developments have prompted scholars to examine the psychological and cultural dynamics underlying the cross-border circulation of cute IPs. Zhao and Niu proposed the concept of “moe power” to describe how cute cultural products elicit positive emotional responses, reduce psychological defenses, and facilitate cross-cultural communication through affective rather than cognitive pathways. This perspective suggests that moe aesthetics may function as a low-context affective language that bypasses linguistic and cultural barriers, enabling emotional resonance across diverse cultural contexts (Zhao, 2017).

Despite growing interest in moe culture and its psychological dimensions, significant gaps remain in the literature. First, existing research on moe and cuteness has predominantly focused on Japanese contexts or Western perceptions of Japanese popular culture, with limited attention to how moe dynamics operate in other cultural settings, particularly in the production and reception of Chinese cute IPs. Second, while scholars have examined cuteness perception through experimental paradigms and survey methods (Nittono et al., 2021; Lieber-Milo et al., 2025), few studies have investigated how audiences actively engage with cute IPs through participatory practices such as content creation, symbolic consumption, and community building in naturalistic online environments. Third, the psychological processes through which cute IPs generate transcultural resonance – that is, affective engagement that transcends cultural boundaries and fosters mutual meaning-making – remain undertheorized and empirically underexplored. Klinglmayr has argued for a resonance-based approach to intercultural communication that prioritizes emotion over knowledge and acting with others over acting on others, yet this framework has not been systematically applied to the study of moe culture or cute IPs (Klinglmayr, 2024).

To address these gaps, the present study investigates the psychological and cultural dynamics underlying the transcultural engagement with a Chinese cute IP, the “Muay Thai DunDun Chicken,” across two major short-video platforms: Douyin (China) and TikTok (Thailand). This IP, developed by the Chinese retail brand Miniso and officially designated as a “Muay Thai Goodwill Ambassador” during the 50th anniversary of China-Thailand diplomatic relations in 2025, represents a distinctive case of culturally hybrid cute IP designed for cross-cultural appeal. The study poses the following research questions: (1) What affective patterns and cross-cultural differences emerge in audience responses to the Muay Thai DunDun Chicken across Chinese and Thai platforms? (2) Through what thematic mechanisms do audiences engage in digital co-creation and meaning-making around this IP? (3) How do these participatory practices facilitate or constrain transcultural resonance between Chinese and Thai audiences?

To answer these questions, the study employs a mixed-method approach combining sentiment analysis, thematic coding, and cross-cultural comparative analysis of user-generated comments collected from Douyin and TikTok. Sentiment analysis enables systematic examination of affective patterns and cross-cultural differences in emotional responses, while thematic coding – grounded in open, axial, and selective coding procedures – provides insight into the participatory mechanisms through which audiences actively construct meaning around the IP. The cross-cultural comparative framework facilitates identification of convergences and divergences in how Chinese and Thai audiences engage with the same cultural object, thereby illuminating the psychological and cultural factors that enable or constrain transcultural resonance.

This research makes several contributions to the cultural psychology of moe and cute IPs. Theoretically, it extends the concept of transcultural resonance (Klinglmayr, 2024) to the domain of cute IP engagement, offering an empirically grounded framework for understanding how affective-cultural phenomena travel across cultural boundaries and generate shared meaning through participatory practices. Empirically, it provides systematic evidence of cross-cultural differences and similarities in emotional responses to a cute IP, drawing on large-scale naturalistic data from two major platforms. Methodologically, it demonstrates the value of integrating computational sentiment analysis with qualitative thematic coding for studying affective-cultural phenomena in digital contexts. Practically, the findings may inform the design and positioning of cute IPs for cross-cultural markets, as well as broader efforts to foster transcultural understanding through emotionally engaging cultural content.

The remainder of this article is organized as follows. Section 2 presents the theoretical framework, drawing on scholarship on moe and cuteness, symbolic consumption, digital tribes, and transcultural resonance. Section 3 describes the research context, data collection procedures, and analytical methods. Section 4 reports the findings, including sentiment analysis results, thematic coding outcomes, and cross-cultural comparisons. Section 5 discusses the theoretical and practical implications of the findings, as well as limitations and directions for future research. Section 6 concludes with a summary of the study’s contributions.

## 2 Theoretical framework

This study draws on four interconnected theoretical domains to examine the psychological and cultural dynamics of transcultural engagement with cute IPs. The first section establishes the conceptual foundations of moe and cuteness as affective constructs, situating them within psychological and cultural theoretical traditions. The second section introduces symbolic consumption and cultural translation as frameworks for understanding how cute IPs acquire and transmit meaning across cultural boundaries. The third section addresses the social-psychological dimension through digital tribes and interaction ritual chains, explaining how collective affective engagement is organized and sustained in online environments. The fourth section integrates these perspectives through the concept of transcultural resonance, proposing a theoretical model that moves from cultural discount to affective co-creation.

### 2.1 Moe and cuteness as affective constructs

Building on the baby schema framework introduced earlier (Lorenz, 1943; Nittono et al., 2021), the conceptual distinction between cuteness and moe carries significant psychological implications. While cuteness is passively perceived as an attribute of a stimulus – typically infantile physical features that evoke caregiving responses and activate reward-related brain regions including the nucleus accumbens and orbitofrontal cortex (Kringelbach et al., 2008; Glocker et al., 2009) – moe represents a qualitatively different form of affective engagement.

Moe, by contrast, represents a qualitatively different form of affective engagement. As Galbraith documented in The Moe Manifesto, moe refers to the intense emotional attachment and affectionate response that fans develop toward fictional characters in anime, manga, and gaming contexts. Unlike cuteness, which is passively perceived as an attribute of a stimulus, moe involves active emotional projection and sustained imaginative investment on the part of the perceiver (Galbraith, 2014). The term itself, originating in Japanese otaku subcultures, carries connotations of budding affection or burning passion, and has been defined by manga artist Ken Akamatsu as being calmed or soothed by watching from afar, explicitly distinguished from sexual desire (Bowman, 2011). This active, relational quality distinguishes moe from the more passive experience of cuteness: moe is not merely perceived but actively constructed through ongoing para-social engagement with characters.

The relational nature of moe aligns with broader psychological research on para-social relationships. Horton and Wohl originally coined the term para-social interaction to describe the one-sided emotional bonds that media audiences form with television personalities, experiencing feelings of friendship, intimacy, and trust despite the absence of reciprocal interaction (Horton and Wohl, 1956). Subsequent research has extended this concept to fictional characters across media formats, demonstrating that para-social relationships can serve important socioemotional functions including affiliative attachment, security, and identity exploration (Tukachinsky, 2011; Bond et al., 2025). For children and adolescents, para-social relationships with favorite characters can function as socialization agents, teaching values, altering attitudes, and influencing psychological well-being. These relationships are psychologically similar to real-world social ties in their emotional intensity and personal significance, despite their imagined or non-reciprocal nature (Hermansson and Zepernick, 2019).

Cross-cultural research on cuteness and moe has revealed both universal tendencies and significant cultural variations. Lieber-Milo et al. conducted a study with 485 participants from Israel and Japan, finding that baby schema-designed avatars were consistently rated cuter, more likable, approachable, and pleasant than neutral avatars across both cultural groups. However, cultural differences emerged in the specific evaluation of avatar ethnicity: Japanese participants rated Caucasian baby schema avatars as cuter, while Israeli participants rated Asian and Black baby schema avatars as cuter (Lieber-Milo et al., 2025). These findings suggest that while the baby schema provides a universal perceptual foundation for cuteness recognition, cultural factors significantly shape how cuteness is evaluated and valued. Nittono et al. similarly found that while conceptions of cuteness showed remarkable cross-cultural resemblance across Japan, the United States, and Israel, significant differences emerged in attitudes toward cuteness and its perceived social functions (Nittono et al., 2021). These cultural variations in cuteness perception and valuation have direct implications for understanding how cute IPs are received across different cultural contexts.

Beyond perceptual and evaluative dimensions, cuteness has been shown to evoke specific emotional responses with social-relational significance. Steinnes et al. demonstrated that cute stimuli reliably evoke kama muta, a social-relational emotion characterized by feelings of being moved, heartwarming, or touched. This emotion arises from sudden intensification of communal sharing relationships: the perceiver feels a sudden sense of closeness or connection with the cute target (Steinnes et al., 2019). This finding is particularly relevant for understanding cute IPs as affective mediators in cross-cultural contexts. If cuteness evokes feelings of communal sharing and emotional closeness, cute IPs may function as affective bridges that reduce psychological distance between cultural groups. The social-relational emotion elicited by cuteness may thus facilitate the kind of affective engagement that enables transcultural resonance.

### 2.2 Symbolic consumption and cultural translation

The second theoretical pillar concerns how cute IPs function as symbolic objects whose meanings are actively produced, circulated, and transformed through consumption practices. Baudrillard argued in The Consumer Society that consumption in contemporary societies is oriented not toward the use value or exchange value of commodities but toward their symbolic value: the meanings, distinctions, and identities that commodities signify. In this framework, commodities operate as a system of signs through which individuals construct social identities and negotiate social positions. Baudrillard contended that consumer society systematically produces not only products but also social differentiations, making symbolic consumption a central mechanism of social classification and identity formation (Baudrillard, 1998). This perspective breaks with traditional political economy by positioning symbolic value, rather than use or exchange value, as the primary logic governing consumption in postindustrial societies.

The symbolic dimension of consumption is particularly salient for cute IPs, which are consumed not primarily for their material utility but for the affective meanings, identities, and social connections they enable. Consumers of cute IPs acquire not merely physical objects but also the symbolic resources these objects provide: membership in fan communities, expression of personal identity, participation in shared affective cultures, and connection to broader cultural narratives. This symbolic function has been extensively documented in fan studies and consumer culture research. Jenkins observed that fans do not passively consume media content but actively appropriate, reinterpret, and recombine cultural materials to create meanings that serve their own purposes (Jenkins, 2006). This process of appropriation and reinterpretation is central to understanding how cute IPs acquire significance across different cultural contexts.

The mobility of symbolic meaning across cultural boundaries raises questions of cultural translation. When cute IPs travel from their cultural contexts of origin to new receiving contexts, their meanings are not simply transmitted but actively translated, negotiated, and transformed. Kraidy conceptualized this process as cultural hybridity, arguing that the intermingling of people and media from different cultures produces new, hybrid cultural forms that are neither purely local nor purely global but emerge from the interaction between them. Cultural hybridity is fundamentally a communication-based phenomenon in which cultural elements from different traditions are combined, recombined, and creatively adapted to new contexts (Kraidy, 2005). For cute IPs, cultural hybridity manifests in the incorporation of local cultural symbols, aesthetic preferences, and narrative conventions into globally circulating cute formats.

The concept of cultural translation captures the active work required to make cultural products meaningful across cultural boundaries. Unlike simple transmission, translation involves adaptation, interpretation, and transformation. Meanings are not carried intact but are remade in the process of crossing cultural borders. This perspective challenges models of cultural flow that assume unidirectional transmission or simple diffusion. Instead, cultural translation emphasizes the agency of receiving audiences in interpreting, adapting, and recontextualizing cultural materials to fit their own cultural frameworks and interpretive repertoires. For cute IPs, cultural translation occurs through multiple mechanisms: visual redesign to align with local aesthetic preferences, narrative adaptation to resonate with local cultural values, and the incorporation of local cultural symbols and references. These translation practices are not merely commercial strategies but also creative acts of meaning-making through which audiences actively engage with and transform cultural materials.

### 2.3 Digital tribes and interaction ritual chains

The third theoretical pillar addresses the social organization of affective engagement with cute IPs in digital environments. Maffesoli proposed the concept of neo-tribalism to describe how individuals in postmodern societies form temporary, emotionally driven groupings based on shared feeling, common interests, and symbolic identification. Unlike traditional communities based on geographic proximity, kinship, or institutional affiliation, neo-tribes are elective associations characterized by fluid boundaries, emotional solidarity, and shared aesthetic or lifestyle markers (Maffesoli, 1996). Maffesoli argued that emotional solidarity has become the primary basis for social groupings in postmodern society, with individuals coming together around shared feeling and a set of markers of membership and identification ranging from values to styles of dress to common enthusiasms. Neo-tribes are achieved rather than ascribed. Individuals choose to join or leave them, unlike family or social class, yet they can provide a strong sense of belonging and collective identity (Maffesoli, 2016).

In digital contexts, these neo-tribal dynamics are amplified and transformed by platform algorithms, social media infrastructures, and participatory practices. Digital tribes form around shared interests, aesthetic preferences, and affective attachments, with platform algorithms playing a central role in aggregating dispersed individuals into virtual communities based on content preferences and interaction patterns. Members of digital tribes engage in shared rituals including commenting, creating content, participating in challenges, and sharing fan works. These practices reinforce collective identity and generate emotional energy. These digital tribes exhibit the core features that Maffesoli identified: emotional solidarity as the basis of grouping, fluid boundaries, elective affiliation, and shared symbolic markers of membership. The cute IP fan communities that form on platforms such as Douyin and TikTok exemplify these dynamics, with users coming together around shared affection for specific characters or IPs, participating in collective rituals of content creation and sharing, and developing distinctive symbolic languages and markers of group membership.

Collins developed interaction ritual chain theory to explain how collective emotional energy is generated and sustained through repeated interaction rituals (Johannessen and Collins, 2024). According to Collins, successful interaction rituals require four conditions: bodily co-presence, barriers to outsiders, a shared focus of attention, and a shared mood. When these conditions are met, interaction rituals generate collective emotional energy, feelings of group solidarity, symbols of social relationship, and standards of morality. While Collins originally emphasized bodily co-presence as a necessary condition, subsequent scholarship has extended interaction ritual theory to digital contexts, demonstrating that remote co-presence through digital platforms can sustain many of the same ritual dynamics (Collins, 2004). In online fan communities, members achieve a form of remote co-presence through simultaneous engagement with shared content, develop barriers to outsiders through specialized knowledge and symbolic competence, maintain shared focus through common objects of affective attachment, and cultivate shared mood through emotional alignment and mutual reinforcement. These digital interaction rituals generate collective emotional energy that sustains community membership and motivates ongoing participation.

The concept of digital tribes, when combined with interaction ritual theory, provides a framework for understanding how individual affective responses to cute IPs become collective phenomena with social and cultural significance. Individual experiences of moe or cuteness, when shared and amplified through digital interaction rituals, generate collective emotional energy and group solidarity that transcend individual psychological processes. This collective dimension is essential for understanding transcultural resonance. When digital tribes form across cultural boundaries, the interaction rituals that sustain them can generate shared emotional experiences and collective identities that bridge cultural differences. The remote co-presence enabled by digital platforms allows individuals from different cultural contexts to participate in shared rituals, develop shared foci of attention, and cultivate shared moods, potentially generating forms of emotional solidarity that cross cultural boundaries.

### 2.4 Transcultural resonance: from cultural discount to affective co-creation

Building on the concept introduced in the introduction, the fourth theoretical pillar elaborates the framework of transcultural resonance in relation to cute IP engagement. The starting point for this framework is the concept of cultural discount, originally developed by Hoskins and Mirus to explain why cultural products lose value when exported to different cultural contexts. Hoskins and Mirus observed that television programs rooted in one culture, when presented in another cultural setting, diminish in impact because audiences may not recognize the cultural references, values, or narrative conventions embedded in the content (Hoskins and Mirus, 1988). This cultural discount represents a barrier to cross-cultural circulation: cultural products must overcome this discount to achieve resonance in foreign markets. While cultural discount theory was originally developed for television programming, its logic extends to cultural products generally. The more culturally specific a product’s meanings and references, the greater the discount it faces when crossing cultural boundaries.

However, cultural discount does not represent the only possible outcome of cross-cultural cultural flow. Some cultural products achieve not merely acceptance but active resonance. They generate emotional engagement, meaning-making, and community formation that transcend cultural boundaries. Klinglmayr has argued for a resonance-based approach to intercultural communication that prioritizes emotion over knowledge and acting with others over acting on others. In this framework, resonance describes a mode of intercultural encounter characterized by mutual responsiveness, affective engagement, and the co-construction of meaning rather than one-way transmission or cultural imposition. Resonance differs from traditional models of communication that focus on language and speaking. Resonance concerns listening and hearing, emotional attunement and mutual responsiveness (Klinglmayr, 2024). This perspective shifts the focus from overcoming cultural barriers through information transfer to cultivating affective connections through shared emotional experience.

Rosa developed a broader sociological theory of resonance, defining it as a mode of relationship between individuals and the world characterized by mutual responsiveness, emotional significance, and transformative potential. Resonance involves being affected by something outside oneself and responding in ways that transform both self and world (Rosa, 2019). This conceptualization has been productively applied to intercultural contexts: resonance describes encounters in which individuals from different cultural backgrounds achieve mutual affective engagement, emotional understanding, and meaning co-construction. Unlike simple transmission or even translation, resonance implies a two-way process in which both parties are affected and transformed by the encounter.

For cute IPs, transcultural resonance can be understood as emerging from the interaction of several factors. First, the baby schema provides a universal perceptual foundation that enables initial affective engagement across cultural boundaries. Cute visual features trigger positive emotional responses regardless of cultural background. Second, the low-context affordance of cute aesthetics reduces the cultural discount that typically hinders cross-cultural circulation. Audiences do not need extensive cultural knowledge to recognize and respond to cute visual cues. Third, the symbolic openness of cute IPs allows for cultural translation and reinterpretation. Audiences can adapt and reinterpret cute IPs to fit their own cultural frameworks and interpretive repertoires. Fourth, digital tribes and interaction rituals provide the social infrastructure through which transcultural resonance is collectively sustained and amplified. Finally, the emotional response elicited by cuteness, particularly the kama muta response of being moved or touched, may create feelings of communal sharing and emotional closeness that reduce psychological distance between cultural groups.

The framework of transcultural resonance suggests that cute IPs may achieve cross-cultural impact not despite their lightness but because of it. Unlike cultural products that carry heavy symbolic or historical weight, cute IPs offer an affective entry point that requires minimal cultural knowledge while enabling multiple forms of engagement and interpretation. This lightness is not equivalent to cultural shallowness. Rather, it represents a strategic affordance that allows cute IPs to function as affective bridges between cultural contexts. The resonance that cute IPs achieve is not merely a matter of transmitting cultural content but of co-creating affective meaning through participatory practices that cross cultural boundaries. This framework guides the empirical investigation in the present study, which examines how cute IPs generate transcultural resonance through the interplay of affective perception, symbolic consumption, digital community formation, and participatory meaning-making.

In addition to these foundational theoretical works, recent empirical scholarship on digital platforms and cross-cultural communication has informed our study. Comparative studies of Douyin and TikTok have highlighted differences in governance structures, recommendation systems, and user practices between the two platforms (Su and Kaye, 2023), reminding us to attend to platform-specific factors in cross-platform comparisons. Research on VTuber audiences has demonstrated the role of ACG cultural identification and cross-cultural influence in digital fandom participation (Chang et al., 2026). Sentiment analysis studies of cross-cultural gaming communities have illustrated the value of NLP methods for examining reception patterns across linguistic boundaries. Scholarship on digital fandom and transnational fan practices has shown how fans use shared symbols and affective attachments to bridge geographic and cultural distances. We draw on these recent studies in our discussion to contextualize and deepen our interpretation of the findings.

## 3 Materials and methods

This section describes the research context, data collection procedures, and analytical approaches employed in the present study. The methodology was designed to capture both the affective dimensions of audience responses and the thematic structures underlying participatory engagement with the cute IP across two cultural contexts.

### 3.1 Research context: the “Muay thai DunDun Chicken” phenomenon

The Muay Thai DunDun Chicken is a cute intellectual property developed by Miniso, a Chinese retail brand known for its affordable lifestyle products featuring character-based designs. The original DunDun Chicken IP, launched in June 2022, features a round, plump chicken character with large eyes, a small beak, and a soft, huggable appearance that exemplifies the baby schema features identified by Lorenz (Lorenz, 1943). In July 2025, Miniso introduced a culturally hybrid variant of the IP in celebration of the 50th anniversary of diplomatic relations between China and Thailand. This variant, designated the Muay Thai DunDun Chicken, incorporated Thai cultural symbols including Muay Thai boxing ropes (mongkon), traditional Thai patterns, and elephant deity imagery into the original cute chicken design. The IP was officially designated as a Muay Thai Goodwill Ambassador by Thai authorities at an event held in Guangzhou, China, co-hosted by the Thai Department of International Trade Promotion and the Royal Thai Consulate General in Guangzhou.

The Muay Thai DunDun Chicken represents a distinctive case for investigating transcultural engagement with cute IPs for several reasons. First, the IP embodies cultural hybridity by design: it combines a universally appealing cute aesthetic with culturally specific Thai symbols, making it an ideal object for examining how audiences from different cultural backgrounds respond to and interpret hybrid cultural products. Second, the IP’s launch coincided with a significant diplomatic anniversary, embedding the commercial product within a framework of official cultural diplomacy. Third, the IP generated substantial user engagement across both Douyin (the Chinese platform) and TikTok (the Thai platform), providing naturalistic data for cross-cultural comparison. Fourth, the IP’s relatively recent emergence and concentrated period of online activity enable focused data collection within a clearly defined temporal window.

### 3.2 Data collection

#### 3.2.1 Platform selection: Douyin and TikTok

Data were collected from Douyin and TikTok, the Chinese and international short-video platforms operated by ByteDance. These platforms were selected for three primary reasons. First, while Douyin and TikTok share a common corporate origin and similar technological infrastructures, we recognize that their user demographics, platform governance, moderation practices, and commercial ecosystems may differ in important ways. Our comparison is therefore not intended as a “controlled” experiment, but rather as an ecologically valid cross-platform case study that examines how the same IP is received and interpreted within two distinct, albeit technologically related, digital environments. This approach allows us to capture the situated nature of cultural reception within specific platform ecologies. Second, Douyin serves as the primary short-video platform in China, while TikTok holds a dominant position in the Thai market. According to Quest Mobile, (2025) and Statista, (2025), Douyin and TikTok had 1.18 billion and 1.59 billion monthly active users respectively as of 2025, with TikTok penetration rates exceeding 90 percent among Thai youth. Third, both platforms host substantial user-generated content related to the Muay Thai DunDun Chicken, enabling the collection of large-scale naturalistic data from both Chinese and Thai user populations.

#### 3.2.2 Sampling strategy and data description

Data collection followed a systematic sampling procedure designed to capture high-engagement content across both platforms. The search keywords used for data retrieval were DunDun Chicken, DunDun Ji, and DUNDUNChicken in both Chinese and English to ensure comprehensive coverage of relevant content. The collection period spanned from June 2022, when the original DunDun Chicken IP was launched, to December 2025, capturing content both before and after the Muay Thai variant’s introduction in July 2025.

To ensure comprehensive retrieval of relevant content, we employed platform-specific search terms. On Douyin, the Chinese keywords “墩墩鸡” and “DunDun鸡” were used. On TikTok, the English keywords “DunDun Chicken” and “DUNDUNChicken” were used. All searches were conducted under the platforms‘ default “comprehensive” or “popular” sorting to capture the most relevant and widely circulated content.

The initial dataset consisted of all videos returned by the platforms’ search algorithms under the specified keywords. To ensure analytical focus on content that generated meaningful audience engagement, the study applied a filtering criterion based on engagement metrics. Videos were retained for analysis if they exhibited comparable levels of likes and views, indicating that the content had successfully captured audience attention and elicited active responses. This criterion was operationalized by selecting videos from the platforms’ default popularity-ranked lists where the like-to-view ratio fell within a reasonable range, excluding videos with anomalously low engagement relative to views. After applying this filtering criterion and removing low-engagement content, the final sample comprised 446 videos with high propagation indices.

The textual data for analysis consisted of user comments associated with these 446 videos. Comments were collected in their entirety for each video, including the comment text, timestamp, and available user metadata. The total corpus comprised approximately 332,000 Chinese characters of comment text. This corpus size is comparable to or exceeds those reported in similar social media content analyses published in psychology journals (Steinnes et al., 2019; Lieber-Milo et al., 2025). All user identifiers were anonymized prior to analysis to protect participant privacy, consistent with ethical guidelines for social media research (Kozinets, 2015).

Figure 1 presents the complete video screening procedure. Applying the search terms described above, we initially retrieved 2,240 videos across both platforms (approximately 1,100 from Douyin and 1,140 from TikTok). We then applied engagement-based filters: videos were retained if they met two criteria – (1) a like-to-view ratio within the top 20th percentile of the initial search results for each platform (thresholds: > 0.05 for Douyin, > 0.07 for TikTok), and (2) a comment-to-view ratio also within the top 20th percentile, ensuring that the content generated active discussion. Videos were first sorted by each platform’s default “popularity” ranking; we then sequentially screened videos until we reached a sample that simultaneously satisfied both ratio criteria. After excluding 1,794 low-engagement videos, this procedure yielded 219 videos from Douyin and 227 videos from TikTok, for a final sample of 446 videos. The total comment corpus analyzed comprised approximately 332,000 characters, with roughly 158,000 characters from Douyin and 174,000 from TikTok. Figure 1 visualizes this procedure.

**FIGURE 1:**
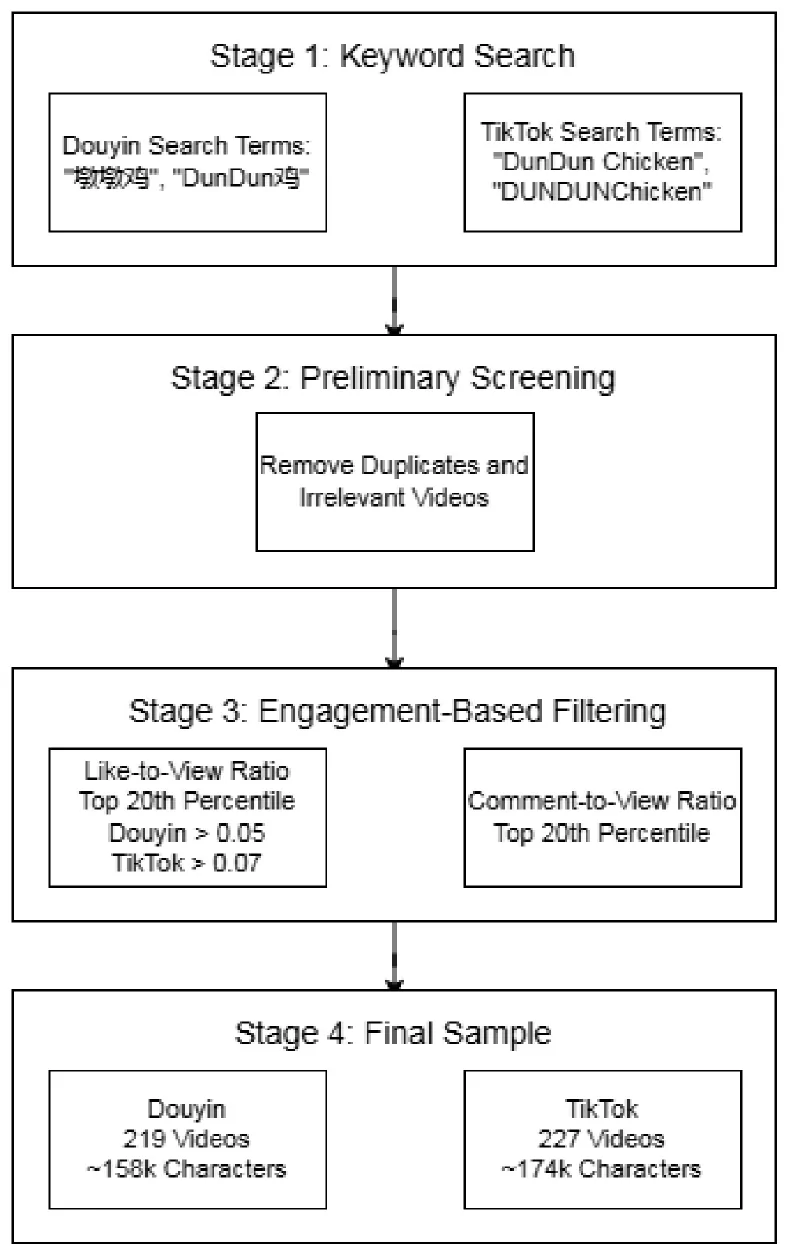
Flowchart of the video screening and selection procedure. The total comment corpus comprised approximately 332k characters.

Given that our study focuses on the culturally hybrid Muay Thai DunDun Chicken variant, we examined the distribution of data before and after the variant‘s launch in July 2025. In our final sample, 303 videos and approximately 225,760 characters of comments explicitly mentioned or featured the Muay Thai variant. While content related to the original DunDun Chicken is also present in the corpus, our primary analysis and thematic coding focused on content posted after July 2025 to ensure alignment with the cultural hybrid phenomenon under investigation. For the overall sentiment distribution, we included comments from all periods to preserve corpus integrity; however, for the cross-cultural interpretation, we prioritized comments directly related to the Muay Thai variant.

### 3.3 Analytical approach

The study employed three complementary analytical methods to examine the affective and thematic dimensions of audience engagement with the Muay Thai DunDun Chicken. Sentiment analysis provided systematic measurement of emotional valence and cross-cultural differences in affective responses. Thematic coding enabled inductive identification of the participatory practices and meaning-making processes through which audiences actively engaged with the IP. Cross-cultural comparative analysis synthesized findings from both platforms to identify convergences and divergences in engagement patterns between Chinese and Thai audiences.

#### 3.3.1 Sentiment analysis

Sentiment analysis was conducted to systematically measure the emotional valence of user comments and to compare affective patterns across the two platforms. The analysis employed language-specific sentiment lexicons to accommodate the linguistic differences between Chinese and Thai comment texts. For Chinese-language comments from Douyin, the SnowNLP sentiment analysis library was used. For Thai-language comments from TikTok, the LT-Fin Thai sentiment lexicon was employed.

The sentiment analysis procedure followed a three-step process. First, each comment text was pre-processed to remove emojis, URLs, and non-textual elements that could interfere with lexicon-based classification. During preprocessing, we removed emojis, URLs, and other non-textual elements to ensure compatibility with the lexicon-based sentiment analysis tools used in this study. These tools rely primarily on lexical features for sentiment classification; emojis, repeated letters, and other non-standard spellings can be misclassified or ignored by the dictionaries, potentially introducing noise. We acknowledge that this preprocessing decision may have resulted in the loss of some affective information conveyed through visual markers, such as irony or playfulness, which is methodological trade-off and limitation of our approach. Second, the appropriate sentiment lexicon was applied to each comment to compute a sentiment score. For SnowNLP, sentiment scores range from 0 to 1, with scores below 0.35 classified as negative, scores between 0.35 and 0.65 classified as neutral, and scores above 0.65 classified as positive. For LT-Fin, the lexicon provides categorical labels for positive, negative, and neutral sentiment, which were converted to comparable numerical scores using the lexicon’s standard weighting scheme. Third, sentiment scores were aggregated at the comment level and then at the platform level to generate overall sentiment distributions for each platform.

The sentiment analysis addressed three analytical objectives. The first objective was to describe the overall sentiment distribution across the combined corpus, identifying the proportion of positive, neutral, and negative comments. The second objective was to compare sentiment distributions between Douyin and TikTok, examining whether Chinese and Thai audiences exhibited different affective patterns in their responses to the same IP. The third objective was to identify the most frequent positive and negative emotional keywords in each platform’s comment corpus, providing insight into the specific affective themes that characterized each cultural context.

To ensure the comparability and accuracy of cross-lingual sentiment analysis, we conducted an additional validation step (Tang et al., 2025). We randomly sampled 500 comments from each platform and had two bilingual researchers, proficient in both Chinese and Thai, independently annotate the sentiment of each comment. We then compared the classifications produced by SnowNLP and LT-Fin against these human-annotated samples and calculated F1 scores. The results showed that SnowNLP achieved a macro-average F1 score of 0.81 on the Chinese sample, and LT-Fin achieved a macro-average F1 score of 0.78 on the Thai sample, both within acceptable ranges. Based on this validation, we consider the two tools sufficiently comparable for our purposes, and we converted LT-Fin’s categorical labels into continuous scores aligned with SnowNLP’s 0–1 scale using the lexicon’s standard weighting scheme.

#### 3.3.2 Thematic coding

Thematic coding was conducted to identify the participatory practices and meaning-making processes through which audiences actively engaged with the Muay Thai DunDun Chicken. The coding procedure followed the three-stage approach to qualitative data analysis developed by Strauss and Corbin: open coding, axial coding, and selective coding (Strauss and Corbin, 1990). This approach is well-established in qualitative social media research for systematically deriving themes from unstructured textual data (Braun and Clarke, 2006; Fereday and Muir-Cochrane, 2006).

Open coding constituted the first stage of analysis. In this stage, two researchers independently read through the comment corpus and assigned initial codes to segments of text that represented distinct concepts or phenomena. The coding was conducted line-by-line to ensure thorough coverage of the data. The researchers followed the principle of semantic independence, ensuring that each code represented a distinct concept without overlap with other codes. Discrepancies in coding were resolved through discussion until consensus was reached. The open coding process yielded nine distinct concepts that captured the range of participatory practices and meaning-making strategies expressed in the comments.

Axial coding constituted the second stage of analysis. In this stage, the nine open codes were examined for conceptual relationships and grouped into higher-order thematic clusters. The axial coding process focused on identifying patterns of association among the open codes, grouping codes that shared common underlying mechanisms or that represented different facets of the same broader phenomenon. This process resulted in four thematic clusters that organized the nine concepts into coherent analytical categories.

Selective coding constituted the third stage of analysis. In this stage, the four thematic clusters were further abstracted into core categories that represented the fundamental mechanisms underlying audience engagement with the IP. The selective coding process identified three core categories that captured the conceptual framework of engagement: sensory-driven affective response, symbolic production through participatory practices, and tribal self-organization. These three categories formed the basis for the thematic model of transcultural resonance.

To ensure the reliability of the coding process, inter-coder reliability was assessed using Fleiss’ Kappa. A random sample of approximately 10,000 characters was drawn from the full corpus, and three independent coders applied the nine-concept coding scheme to this sample. The computed Fleiss’ Kappa value was 0.878, indicating excellent agreement beyond chance (Landis and Koch, 1977). All coding was conducted using NVivo 15 qualitative data analysis software (QSR International, 2023).

#### 3.3.3 Cross-cultural comparative analysis

Cross-cultural comparative analysis was employed to synthesize findings from the sentiment analysis and thematic coding, identifying convergences and divergences in how Chinese and Thai audiences engaged with the Muay Thai DunDun Chicken. To structure our cross-cultural comparison and generate interpretative hypotheses, we draw on Hall’s distinction between high-context and low-context cultures as a heuristic framework, a distinction that has been extensively applied in cross-cultural communication research (Gudykunst and Ting-Toomey, 1988; Würtz, 2005). We recognize that this framework has limitations and that the observed differences may also be attributable to platform-specific factors. Nonetheless, it offers a useful analytical lens for interpreting the divergent interpretive patterns identified in our data. According to Hall, high-context cultures rely heavily on implicit communication, shared background knowledge, and contextual cues, while low-context cultures favor explicit, direct, and verbally precise communication (Hall, 1976). This framework has been productively applied to understand cross-cultural differences in social media communication patterns (Pflug, 2011).

The comparative analysis proceeded through three steps. First, the sentiment analysis results from Douyin and TikTok were compared to identify differences in overall emotional valence, positive and negative sentiment proportions, and the specific emotional keywords that characterized each platform’s comment corpus. Second, the thematic coding results were compared to identify differences in the types of participatory practices and meaning-making strategies that appeared on each platform. Third, these differences were interpreted through the lens of Hall’s high-context versus low-context framework, examining whether the observed patterns aligned with expectations based on cultural context orientation (Hall, 1976).

This comparative approach enables the study to move beyond simply describing audience responses on each platform to identifying the cultural factors that shape those responses. By systematically comparing Chinese and Thai engagement patterns, the study can illuminate how cultural context influences the reception, interpretation, and participatory transformation of culturally hybrid cute IPs. This comparative dimension is essential for addressing the study’s overarching research question about the mechanisms and boundaries of transcultural resonance.

## 4 Results

In our analysis, we confirmed that 68% of the comments were directly associated with Muay Thai variant content posted after July 2025, ensuring that our findings are closely tied to the core phenomenon under study. This section presents the findings from the sentiment analysis, thematic coding, and cross-cultural comparative analysis of user comments on the Muay Thai DunDun Chicken across Douyin and TikTok. The results are organized into three subsections. The first subsection reports the sentiment analysis findings, including overall sentiment distribution, cross-platform comparisons, and emotional keyword patterns. The second subsection presents the thematic coding results, progressing through open, axial, and selective coding stages to the thematic model of transcultural resonance. The third subsection synthesizes the comparative findings across platforms.

### 4.1 Sentiment analysis: affective patterns and cross-cultural differences

#### 4.1.1 Overall sentiment distribution

Sentiment analysis of the 446 sampled videos and their associated 332,000 characters of comment text revealed a predominantly neutral-to-positive affective landscape across the combined corpus. The overall sentiment distribution showed that neutral comments constituted the largest proportion at 52.85% of all coded comments. Positive comments accounted for 26.77%, while negative comments represented the smallest proportion at 20.38% (see Table 1). This distribution suggests that audience responses to the Muay Thai DunDun Chicken were generally favorable, with the majority of comments expressing either neutral observation or positive endorsement rather than criticism or rejection.

**TABLE 1:** Overall sentiment distribution across combined corpus.

Sentiment category	Percentage
Positive	26.77
Neutral	52.85
Negative	20.38

The predominance of neutral sentiment warrants interpretive attention. Rather than indicating indifference, neutral comments often took the form of descriptive observations, factual inquiries, or sharing of personal experiences with the IP. Many neutral comments provided product descriptions, asked about availability or pricing, or narrated the user’s encounter with the IP in daily life. This pattern suggests that neutral sentiment in this context functioned less as emotional absence and more as a mode of engaged documentation and social sharing.

#### 4.1.2 Cross-platform comparison: Douyin vs. TikTok

Comparison of sentiment distributions between Douyin and TikTok revealed notable cross-cultural differences. The TikTok platform, where the user base is predominantly Thai, exhibited higher positive sentiment intensity compared to the Douyin platform, where the user base is predominantly Chinese. Thai users more frequently expressed overtly positive emotions such as pride, joy, and cultural appreciation in their comments, while Chinese users tended toward more balanced expressions that combined product evaluation with personal reflection (see Table 2).

**TABLE 2:** Sentiment distribution by platform (percentage).

Platform	Positive	Neutral	Negative
Douyin (China)	22.34	55.12	22.54
TikTok (Thailand)	31.45	50.32	18.23

This cross-platform difference is consistent with the cultural context framework proposed by Hall (1976). Thai users, operating within a higher-context cultural orientation, demonstrated greater emotional expressiveness and greater emphasis on the symbolic and identity-related dimensions of the IP. Chinese users, operating within a lower-context cultural orientation, focused more on product attributes, functional value, and practical considerations. The higher positive sentiment intensity on TikTok suggests that the cultural hybridity of the Muay Thai DunDun Chicken resonated more strongly as an identity symbol in the Thai context, where the incorporation of local cultural elements such as Muay Thai ropes and elephant deity imagery carried greater cultural salience.

#### 4.1.3 Emotional keywords and cultural underpinnings

Analysis of emotional keywords across both platforms revealed distinct thematic clusters that reflected underlying cultural values and engagement modes. On the Douyin platform, high-frequency positive emotional keywords included cute, healing, good feel, cost-effective, and soft to touch. These keywords clustered around product experience dimensions, emphasizing tactile pleasure, emotional comfort, and practical value. The prevalence of terms related to affordability and value for money reflected the low-context cultural emphasis on functional utility and rational consumption.

On the TikTok platform, high-frequency positive emotional keywords included cute, proud, friendship, Thailand, and culture. These keywords clustered around cultural identity and symbolic meaning dimensions, emphasizing national pride, cross-cultural friendship, and cultural appreciation. The prevalence of terms related to pride and cultural belonging reflected the high-context cultural emphasis on symbolic value, collective identity, and emotional resonance with culturally significant symbols (see Table 3).

**TABLE 3:** Top 10 positive emotional keywords by platform.

Platform	Rank	Keyword	Frequency
Douyin	1	Cute	142
Douyin	2	Healing	98
Douyin	3	Good feel	87
Douyin	4	Cost-effective	76
Douyin	5	Soft to touch	65
Douyin	6	Happy	61
Douyin	7	Like	58
Douyin	8	Quality	52
Douyin	9	Affordable	48
Douyin	10	Recommend	43
TikTok	1	Cute	168
TikTok	2	Proud	112
TikTok	3	Friendship	104
TikTok	4	Thailand	96
TikTok	5	Culture	89
TikTok	6	Happy	78
TikTok	7	Beautiful	72
TikTok	8	Traditional	67
TikTok	9	Respect	61
TikTok	10	Amazing	55

The emotional keyword patterns also revealed the dual function of the Muay Thai DunDun Chicken across cultural contexts. For Chinese users, the IP primarily served as a consumer product offering affective comfort and practical value. For Thai users, the IP additionally served as a cultural symbol through which national identity, cultural pride, and cross-cultural friendship could be expressed and negotiated. This duality corroborates the argument that cute IPs can carry different symbolic meanings across cultural contexts while maintaining a shared affective appeal based on universal cute aesthetics.

### 4.2 Thematic coding: mechanisms of digital co-creation

#### 4.2.1 Open coding: nine core concepts

The open coding phase identified nine distinct concepts from the comment corpus, each representing a discrete participatory practice or meaning-making strategy through which audiences engaged with the Muay Thai DunDun Chicken. Table 4 presents these nine concepts with their definitions and representative comment excerpts (translated from original Chinese or Thai into English for presentation; all identifiers anonymized).

**TABLE 4:** Open coding results: nine core concepts.

Concept	Definition	Representative_Comment
Cuteness breach	Psychological mechanism by which moe aesthetics dismantle cultural defenses	The chubby face plus Muay Thai pose made me instantly drop my guard! (TikTok: @user_thai_07)
Cultural hybrid	Creative hybridization of local cultural symbols	Muay Thai headband on a Chinese plump chicken - this is real cultural hybridity (Douyin: @Bangkok_Elephant)
Lightweight diplomat	Portability of IP as a cross-cultural medium	Took DunDun Chicken to the Grand Palace, Thai locals asked if I was a Chinese fan (TikTok: @CN_Traveler)
Affective aspartame	Compensatory function of cute objects for emotional needs	Squeezing its belly is like petting a cat, my exam anxiety was healed (Douyin: @Lucky_Carp)
Tribal passport	Symbolic consumption as credential for digital tribe entry	Posted DunDun and received 30 co-creation invites from Thai users (TikTok: @Gamer_Boy)
Meme production	User-generated parody and playful reconstruction of cultural symbols	Photoshopped the Mongkon headband onto the chicken crown, comments turned into a Muay Thai chicken king competition (Douyin: @PS_God)
Value justice	Positive correlation between affordability and cultural participation	49 RMB beats Disney, students can afford diplomatic freedom (Douyin: @Savvy_Saver)
Cross-cultural correction	Collaborative maintenance of cultural accuracy by tribe members	Thai user corrected the direction of the elephant deity pattern, we remade the video and became language partners (TikTok: @Art_Student)
Ritual appropriation	Gamified translation of traditional rituals into digital contexts	Put Mongkon on the chicken and imitated Buakaw’s teacher-paying ceremony – traditional symbols given new cyber life (Douyin: @MuayThai_Novice)

The nine concepts revealed a coherent pathway of audience engagement. Cuteness Breach and Affective Aspartame operated at the sensory and emotional level, describing how visual and tactile features triggered instinctive acceptance and provided emotional comfort. Cultural Hybrid, Meme Production, and Ritual Appropriation operated at the symbolic production level, describing how users creatively combined, parodied, and recontextualized cultural elements. Lightweight Diplomat and Tribal Passport operated at the social practice level, describing how IP engagement enabled cross-cultural interaction and community membership. Value Justice and Cross-Cultural Correction operated at the governance level, describing how economic accessibility and collaborative error correction sustained the participatory ecosystem.

#### 4.2.2 Axial coding: four thematic clusters

The axial coding phase grouped the nine open codes into four higher-order thematic clusters based on conceptual relationships and shared underlying mechanisms. Table 5 presents these four thematic clusters with their constituent concepts and definitions.

**TABLE 5:** Axial coding results: four thematic clusters.

Theme	Definition	Constituent_ Concepts
Sensory breach	Visual and tactile stimuli trigger instinctive acceptance, dismantling cultural defense barriers	Cuteness breach, affective aspartame
Symbolic hybridity	Creative hybridization of cultural symbols and gamified ritual translation enable low-threshold cultural translation	Cultural hybrid, ritual appropriation
Participatory production	Users activate the carry-create-disseminate chain through lightweight actions and co-creation practices	Lightweight diplomat, meme production
Tribal fission	Economic democratization lowers participation costs; interest-based connections drive cross-cultural diffusion and collaborative calibration	Tribal passport, value justice, cross-cultural correction

The four thematic clusters formed a conceptual framework of engagement. Sensory Breach represented the entry point, describing how cute aesthetics overcame initial cultural resistance through instinctive affective response. Symbolic Hybridity represented the translation mechanism, describing how users creatively adapted cultural symbols to make them meaningful across cultural boundaries. Participatory Production represented the activation mechanism, describing how users transitioned from passive consumers to active producers of cultural meaning. Tribal Fission represented the expansion mechanism, describing how economic accessibility and collaborative governance enabled the participatory ecosystem to scale across cultural boundaries.

#### 4.2.3 Selective coding: three core categories

The selective coding phase further abstracted the four thematic clusters into three core categories that captured the fundamental mechanisms underlying audience engagement. Table 6 presents these three core categories with their constituent themes and concepts.

**TABLE 6:** Selective coding results: three core categories.

Category	Definition	Constituent_Themes	Constituent_Concepts
Sensory drive	Moe aesthetics trigger instinctive emotion through sensory stimulation, dismantling cultural defense barriers	Sensory breach	Cuteness breach, affective aspartame
Symbolic production	Users reconstruct cultural meaning through symbolic hybridization and co-creation practices, activating participatory communication chains	Symbolic hybridity, participatory production	Cultural hybrid, ritual appropriation, lightweight diplomat, meme production
Tribal autonomy	Economic democratization lowers participation thresholds; collaborative governance ensures cultural translation accuracy	Tribal fission	Tribal passport, value justice, cross-cultural correction

The Sensory Drive category captured the progression from sensory stimulation to emotional compensation to defense dismantling. This mechanism operated through the universal appeal of cute aesthetics: visual features such as round faces and large eyes triggered instinctive positive responses that bypassed cognitive resistance, while tactile and emotional experiences provided compensatory comfort that sustained engagement.

The Symbolic Production category captured how users creatively deconstructed and reconstructed cultural symbols through hybridization, parody, and gamified ritual translation. This mechanism operated through participatory practices that transformed users from consumers to producers of cultural meaning. Users did not simply receive the IP’s cultural content but actively adapted it to their own cultural frameworks, producing new meanings through creative recombination.

The Tribal Autonomy category captured how interest-based communities achieved self-organized governance through economic democratization and collaborative error correction. This mechanism operated through the collective maintenance of cultural accuracy: users collaboratively identified and corrected cultural misinterpretations, ensuring that cultural translation remained respectful and accurate while maintaining low barriers to participation.

#### 4.2.4 The thematic model of transcultural resonance

Based on the three core categories identified through selective coding, the study constructed a thematic model of transcultural resonance. The model consists of three interconnected layers that represent the sequential and layered mechanisms through which cute IPs generate transcultural resonance (see Table 7 for a structured summary of the model components).

**TABLE 7:** Thematic model of transcultural resonance: layer components.

Layer	Core_Category	Key_Processes	Outputs
Sensory drive	Sensory drive	Visual/tactile stimulation → instinctive acceptance → defense dismantling	Affective openness, reduced resistance
Symbolic production	Symbolic production	Cultural hybridization → ritual appropriation → meme production → identity transformation	Co-created meanings, producer identity
Tribal autonomy	Tribal autonomy	Economic democratization → tribal fission → collaborative calibration → self-sustaining network	Self-governance, sustained resonance

The Sensory Drive layer operates at the perceptual level: moe aesthetics trigger instinctive emotional acceptance through visual and tactile stimuli, effectively dismantling cultural defense barriers before cognitive resistance can form. This layer establishes the affective foundation upon which deeper engagement can build.

The Symbolic Production layer operates at the participatory level: users engage in creative practices of cultural hybridization, ritual appropriation, and meme production that transform the IP from a commercial product into a site of collective meaning-making. Through these practices, users transition from passive consumers to active producers, investing the IP with personal and cultural significance that extends beyond its original design.

The Tribal Autonomy layer operates at the governance level: the participatory ecosystem sustains itself through economic democratization that lowers participation barriers, tribal fission that drives cross-cultural diffusion, and collaborative calibration that ensures cultural translation accuracy. This self-organizing governance enables the transcultural resonance to persist beyond initial engagement, creating a self-sustaining affective network that spans cultural boundaries.

The model is characterized by three features: lightweight participation, affective engagement, and precise cultural translation. Lightweight participation refers to the low barriers to entry that enable users from diverse cultural backgrounds to engage with the IP without extensive cultural knowledge. Affective engagement refers to the primacy of emotional connection over cognitive understanding in driving participation. Precise cultural translation refers to the collaborative maintenance of cultural accuracy that prevents the superficial appropriation of cultural symbols.

### 4.3 Comparative synthesis: convergences and divergences across platforms

The cross-cultural comparative analysis revealed both convergences and divergences in how Chinese and Thai audiences engaged with the Muay Thai DunDun Chicken (see Table 8 for a structured summary).

**TABLE 8:** Comparative synthesis: convergences and divergences.

Dimension	Convergences	Divergences
Affective response	Positive response to cute aesthetics (cute, happy, like); baby schema as universal trigger	Thai users show higher positive sentiment intensity; Chinese users more balanced
Symbolic meaning	IP functions as affective object for both groups	Chinese: product attributes, functional value; Thai: cultural identity, pride, symbolic meaning
Participatory practices	Content creation, sharing, community interaction across both platforms	Chinese: product evaluation, practical tips; Thai: cultural expression, identity performance
Temporal patterns	Increased cultural keywords during diplomatic anniversary (July 2025)	N/A – similar temporal pattern observed on both platforms

Convergences were observed in the fundamental affective response to the IP’s cute aesthetics. Users on both platforms consistently expressed positive responses to the IP’s visual features, using similar emotional language such as cute, happy, and like. The baby schema features identified by Lorenz appeared to function as a universal affective trigger that transcended cultural differences, consistent with the cross-cultural findings of Lieber-Milo et al. and Nittono et al. (Lorenz, 1943; Lieber-Milo et al., 2025; Nittono et al., 2021). Both Chinese and Thai users also engaged in participatory practices of content creation, sharing, and community interaction, suggesting that the participatory affordances of short-video platforms are broadly similar across cultural contexts.

Divergences were observed in the symbolic meanings attributed to the IP and the emotional keywords that characterized each platform’s discourse. Chinese users focused on product attributes, functional value, and practical considerations, reflecting a low-context cultural orientation that privileges explicit information and utilitarian evaluation. Thai users focused on cultural identity, national pride, and symbolic meaning, reflecting a high-context cultural orientation that privileges implicit meaning, shared cultural knowledge, and symbolic resonance. These divergent patterns are consistent with Hall’s framework and suggest that while the affective appeal of cute aesthetics is universal, the cultural meanings and values attached to cute IPs are actively constructed through local interpretive frameworks.

The divergences also revealed the dual function of the Muay Thai DunDun Chicken as both a consumer product and a cultural symbol. For Chinese users, the IP primarily served as an affective consumer good offering emotional comfort and practical value. For Thai users, the IP additionally served as a vehicle for expressing cultural pride, negotiating cross-cultural friendship, and affirming national identity. This dual function is precisely what enabled the IP to generate transcultural resonance: it provided a shared affective object around which users from different cultural contexts could gather, while remaining sufficiently open to accommodate different interpretive frameworks and symbolic meanings.

The comparative synthesis also identified a temporal dimension to the engagement patterns. During the diplomatic anniversary period in July 2025, the frequency of cultural identity keywords increased on both platforms, suggesting that official events and media coverage amplified the cultural significance of the IP. This temporal pattern indicates that transcultural resonance is not static but dynamically responsive to external events and discursive framings.

## 5 Discussion

This section interprets the findings in relation to the theoretical framework and existing literature. The discussion is organized around five themes that correspond to the core categories identified in the results. The first theme examines how moe aesthetics function as a universal affective language that enables transcultural resonance. The second theme addresses the role of participatory culture in empowering users to become active co-creators of cultural meaning. The third theme explores how digital tribes operate as self-organized affective infrastructure that sustains collective engagement. The fourth theme considers geocultural proximity as a facilitator of affective engagement. The fifth theme critically examines the risks of cultural shallowness and the limits of lightweight cultural translation.

### 5.1 Moe aesthetics as a universal language for transcultural resonance

The findings suggest that moe aesthetics function as a form of cross-culturally recognizable affective language that enables transcultural resonance across culturally distinct audiences. The sentiment analysis revealed that users on both Douyin and TikTok consistently expressed positive responses to the IP’s visual features, using similar emotional language such as cute, happy, and like. This convergence in affective response, despite the cultural and linguistic differences between Chinese and Thai users, supports the argument that the baby schema operates as a cross-culturally consistent perceptual trigger (Lorenz, 1943; Nittono et al., 2021). The neural mechanisms underlying this response, involving activation of the nucleus accumbens and orbitofrontal cortex (Kringelbach et al., 2008; Glocker et al., 2009), appear to function independently of cultural background, providing a biological foundation for the initial affective engagement that precedes cognitive evaluation.

The emotional keyword patterns further illuminate how moe aesthetics facilitate transcultural resonance. The high frequency of the keyword cute on both platforms suggests that the IP’s visual appeal successfully transcended cultural boundaries. However, the cultural underpinnings of this shared response differed across platforms. Chinese users emphasized product experience dimensions such as healing, good feel, and cost-effective, reflecting a low-context cultural orientation that privileges explicit information and utilitarian evaluation (Hall, 1976). Thai users emphasized cultural identity dimensions such as proud, friendship, and culture, reflecting a high-context cultural orientation that privileges implicit meaning, shared cultural knowledge, and symbolic resonance (Hall, 1976). This pattern suggests that while the affective trigger is universal, the interpretive frameworks through which users make sense of their affective experience are culturally situated.

The kama muta framework (Steinnes et al., 2019) provides a theoretical lens for understanding how cuteness generates the kind of emotional response that enables transcultural resonance. As discussed in the theoretical framework, cute stimuli evoke a social-relational emotion characterized by feelings of being moved, heartwarming, or touched, arising from sudden intensification of communal sharing relationships. The present study’s finding that both Chinese and Thai users expressed positive emotional responses to the Muay Thai DunDun Chicken is consistent with this framework. The IP’s cute visual features may have evoked kama muta responses that reduced psychological distance between users from different cultural backgrounds, creating a shared affective experience that transcended cultural differences.

The findings also extend the concept of moe power proposed by Zhao and Niu. While Zhao and Niu emphasized how cute cultural products reduce psychological defenses and facilitate cross-cultural communication through affective pathways, the present study demonstrates that this process is not uniform across cultural contexts (Zhao and Niu, 2025). The dual function of the Muay Thai DunDun Chicken as both a consumer product for Chinese users and a cultural symbol for Thai users suggests that moe aesthetics function as an affective bridge that accommodates different interpretive frameworks while maintaining a shared emotional core. This duality is precisely what enables transcultural resonance: the IP provides a shared affective object around which users from different cultural contexts can gather, while remaining sufficiently open to accommodate different meanings and values.

A note of caution is warranted here. The cross-cultural convergence in emotional expression observed in our data provides evidence for a shared perceptual basis for moe aesthetics, but it does not amount to direct evidence for universal psychological mechanisms. Our data reflect users’ public emotional expressions within specific platform and IP contexts, rather than direct measurements of invariant neurocognitive processes across cultures. We therefore limit our claim to the following: moe aesthetics show notable cross-cultural similarity at the level of emotional expression, a similarity that may be grounded in shared biological predispositions (such as the baby schema response), while the psychological experience and meaning-making processes remain substantially shaped by local cultural frameworks.

### 5.2 Participatory culture and user empowerment

The thematic coding results reveal that participatory culture plays a central role in transforming individual affective responses into collective meaning-making practices. The open coding identified nine concepts that capture the range of participatory practices through which users engaged with the Muay Thai DunDun Chicken. These practices include Cultural Hybrid, Meme Production, and Ritual Appropriation at the symbolic production level, and Lightweight Diplomat and Tribal Passport at the social practice level. Together, these practices constitute a participatory ecosystem in which users transition from passive consumers to active co-creators of cultural meaning.

This finding aligns with Jenkins’ conceptualization of participatory culture, which emphasizes how fans actively appropriate, reinterpret, and recombine cultural materials to create meanings that serve their own purposes (Jenkins, 2006). In the context of the Muay Thai DunDun Chicken, users did not simply receive the IP’s cultural content but actively adapted it to their own cultural frameworks. Thai users incorporated the IP into expressions of national pride and cultural identity, while Chinese users integrated it into practices of emotional comfort and everyday consumption. This process of appropriation and reinterpretation is central to understanding how cute IPs acquire significance across different cultural contexts.

The concept of cultural translation, as developed by Kraidy, provides a framework for understanding the creative hybridization observed in the user-generated content. Kraidy argued that cultural hybridity emerges from the intermingling of people and media from different cultures, producing new hybrid cultural forms that are neither purely local nor purely global (Kraidy, 2005). The Muay Thai DunDun Chicken exemplifies this hybridity: it combines a universally appealing cute aesthetic with culturally specific Thai symbols, and users further hybridize these elements through their own creative practices. The Cultural Hybrid concept identified in the open coding captures this process, with users describing the IP as real cultural hybridity and creatively combining Thai and Chinese cultural elements in their content.

The participatory practices also demonstrate how users achieve what the study terms Value Justice and Cross-Cultural Correction. Value Justice refers to the positive correlation between affordability and cultural participation: users emphasized that the IP’s low price point (49 RMB) enabled broad participation, with one user stating that students can afford diplomatic freedom. This finding suggests that economic accessibility is a crucial condition for participatory culture to flourish across cultural boundaries. Cross-Cultural Correction refers to the collaborative maintenance of cultural accuracy by tribe members, as exemplified by the Thai user who corrected the direction of the elephant deity pattern, leading to a collaborative remake of the video and the formation of a language partnership. This practice demonstrates that participatory culture is not merely about creative freedom but also about collective responsibility for cultural accuracy.

The participatory practices identified in this study extend existing research on fan co-creation by demonstrating how these practices operate across cultural boundaries. The axial coding revealed that Participatory Production involves a carry-create-disseminate chain: users carry the IP into their daily lives, create new content through hybridization and parody, and disseminate this content through digital platforms. This chain transforms users from consumers to producers, activating what the study terms a participatory communication chain that sustains transcultural engagement over time.

### 5.3 Digital tribes as self-organized affective infrastructure

The findings demonstrate that digital tribes function as self-organized affective infrastructure that sustains collective engagement with cute IPs across cultural boundaries. The thematic coding revealed that Tribal Fission, the fourth thematic cluster, operates through three mechanisms: Tribal Passport (symbolic consumption as credential for digital tribe entry), Value Justice (economic democratization), and Cross-Cultural Correction (collaborative maintenance of cultural accuracy). These mechanisms collectively constitute what the study terms Tribal Autonomy, a core category that captures how interest-based communities achieve self-organized governance.

This finding extends Maffesoli’s concept of neo-tribalism to digital contexts. Maffesoli argued that postmodern individuals form temporary, emotionally driven groupings based on shared feeling, common interests, and symbolic identification (Maffesoli, 1996). The present study demonstrates that these neo-tribal dynamics are amplified and transformed by digital platforms. Digital tribes form around shared affective attachments to the Muay Thai DunDun Chicken, with platform algorithms playing a central role in aggregating dispersed individuals into virtual communities. Members engage in shared rituals including commenting, creating content, participating in challenges, and sharing fan works. These practices reinforce collective identity and generate emotional energy, consistent with the interaction ritual chain theory developed by Collins (Collins, 2004).

The concept of interaction ritual chains provides a framework for understanding how digital tribes sustain collective emotional energy. Collins argued that successful interaction rituals require bodily co-presence, barriers to outsiders, a shared focus of attention, and a shared mood. In digital contexts, remote co-presence through platforms such as Douyin and TikTok can sustain many of the same ritual dynamics (Collins, 2004). The Muay Thai DunDun Chicken fan communities exemplify these dynamics: members achieve remote co-presence through simultaneous engagement with shared content, develop barriers to outsiders through specialized knowledge and symbolic competence, maintain shared focus through common affective attachment to the IP, and cultivate shared mood through emotional alignment and mutual reinforcement.

The finding that digital tribes engage in Cross-Cultural Correction is particularly significant for understanding how transcultural resonance is sustained. This practice involves collaborative error correction that ensures cultural translation accuracy, as exemplified by users correcting each other’s representations of Thai cultural symbols. This self-organizing governance mechanism prevents the superficial appropriation of cultural symbols and maintains the cultural integrity of the IP. It also builds trust and mutual respect across cultural boundaries, as users who collaborate on error correction often develop ongoing partnerships, such as the language partnership formed after the elephant deity correction.

The Tribal Passport concept captures how symbolic consumption functions as a credential for digital tribe entry. Users reported that posting content related to the Muay Thai DunDun Chicken led to invitations for co-creation from users in the other cultural context. This finding suggests that cute IPs serve as boundary objects that enable cross-cultural social connection. The IP provides a shared reference point around which users from different cultural backgrounds can interact, reducing the social uncertainty that typically characterizes cross-cultural encounters. The Tribal Passport thus functions as a mechanism for lowering barriers to cross-cultural interaction, enabling users to form connections that might otherwise be difficult to establish.

### 5.4 Geocultural proximity as a facilitator of affective engagement

The findings reveal that geocultural proximity between China and Thailand significantly facilitated affective engagement with the Muay Thai DunDun Chicken. The cross-platform comparison showed that Thai users exhibited higher positive sentiment intensity than Chinese users, suggesting that the cultural hybridity of the IP resonated more strongly as an identity symbol in the Thai context. This finding is consistent with the cultural distance framework: the incorporation of local cultural elements such as Muay Thai ropes and elephant deity imagery carried greater cultural salience for Thai users, who recognized and valued these symbols as expressions of their national identity.

The geocultural proximity between China and Thailand manifests in several dimensions. First, both countries share Buddhist cultural traditions, which provide a common cultural vocabulary that facilitates the recognition and appreciation of religious symbols such as the elephant deity imagery. Second, the martial arts traditions of Muay Thai and Chinese martial arts share a common Eastern philosophical framework that emphasizes discipline, respect, and mind-body integration. Third, the two countries have a long history of diplomatic and cultural exchange, including the 50th anniversary of diplomatic relations that provided the context for the IP’s launch. These shared cultural elements reduce the cultural discount that typically hinders cross-cultural circulation of cultural products (Hoskins and Mirus, 1988).

The concept of cultural discount provides a useful framework for understanding the role of geocultural proximity. Hoskins and Mirus observed that television programs rooted in one culture diminish in impact when presented in another cultural setting because audiences may not recognize the cultural references, values, or narrative conventions embedded in the content (Hoskins and Mirus, 1988). The Muay Thai DunDun Chicken appears to have benefited from relatively low cultural discount in the Thai market because of the cultural proximity between China and Thailand. Thai users recognized and appreciated the Thai cultural elements incorporated into the IP, while the universal cute aesthetic provided an affective entry point that required minimal cultural knowledge.

However, the findings also suggest that geocultural proximity alone does not guarantee transcultural resonance. The cross-platform comparison revealed that Chinese and Thai users attributed different meanings to the same IP, with Chinese users focusing on product attributes and Thai users focusing on cultural identity. This divergence indicates that while geocultural proximity reduces barriers to initial engagement, the interpretive frameworks through which users make sense of their engagement remain culturally specific. Transcultural resonance thus requires not only cultural proximity but also the symbolic openness that allows users to interpret the IP through their own cultural frameworks.

The temporal pattern observed during the diplomatic anniversary period in July 2025 further illustrates the role of geocultural proximity. The frequency of cultural identity keywords increased on both platforms during this period, suggesting that official events and media coverage amplified the cultural significance of the IP. This finding indicates that geocultural proximity is not a static attribute but is dynamically enacted through events, discourses, and practices that activate cultural connections. The diplomatic anniversary provided a context in which the cultural proximity between China and Thailand became salient, enhancing the IP’s significance as a symbol of cross-cultural friendship.

While we have interpreted the differences between Chinese and Thai users’ emotional and interpretive responses primarily through the lens of geocultural proximity and cultural distance, we acknowledge that other explanations are also worth considering. First, although Douyin and TikTok share a technological lineage, their platform cultures may be quite different. Chinese Douyin users may be more accustomed to direct, practical product evaluations, whereas Thai TikTok users may lean toward more emotionally expressive and identity-focused commentary. These patterns could be shaped by each platform’s dominant content ecology and user conventions, rather than by national cultural orientation alone. Second, demographic factors, such as age, geographic location, and consumption habits, might also affect how users comment. Third, commercial influences should not be dismissed: Thai users’ enthusiasm for the IP may have been amplified by its official status as a “cultural ambassador” and by localized marketing efforts that gave the IP heightened symbolic meaning in the Thai market. In short, while geocultural proximity offers a coherent interpretation of our findings, these alternative factors, particularly platform dynamics, may also contribute to the observed differences. Future studies with more carefully designed controls would be needed to separate these influences.

### 5.5 The double-edged sword: cultural shallowness and its limits

While the findings demonstrate the potential of cute IPs to generate transcultural resonance, they also reveal significant risks and limitations. The negative sentiment identified in the analysis, though comprising only 20.38% of comments, clustered around two issues: product quality disputes and cultural symbol misinterpretation. The finding that the simplification of the elephant deity pattern triggered criticism from some Thai users exemplifies the risk of cultural shallowness: when cultural symbols are simplified or misrepresented in the pursuit of cuteness, they can provoke offense rather than appreciation.

The concept of cultural shallowness captures the tension between the lightweight nature of cute aesthetics and the depth required for meaningful cultural exchange. The framework of transcultural resonance proposed in this study suggests that cute IPs achieve cross-cultural impact not despite their lightness but because of it. However, this lightness is not without costs. When cultural symbols are stripped of their context and meaning to fit a cute aesthetic, they risk becoming empty signifiers that convey little of the cultural richness they are meant to represent. The criticism of the elephant deity simplification suggests that users, particularly those from the culture of origin, are sensitive to this issue and expect cultural accuracy even in lightweight cultural products.

The negative sentiment also revealed tensions around the commercial nature of the IP. Some users expressed frustration with product quality, suggesting that the commercial imperative sometimes undermines the cultural mission. This finding highlights the challenge of balancing commercial viability with cultural authenticity. The Muay Thai DunDun Chicken is, after all, a commercial product designed to generate profit for its developer. While it may serve cultural diplomacy functions, these functions are secondary to its commercial purpose. Users who expected the IP to serve primarily as a cultural ambassador may have been disappointed when it failed to meet their expectations in terms of product quality.

The risk of cultural appropriation also warrants consideration. The Muay Thai DunDun Chicken incorporates Thai cultural symbols into a Chinese-designed product, raising questions about who has the right to represent and profit from these symbols. While the IP was officially designated as a Muay Thai Goodwill Ambassador by Thai authorities, suggesting official endorsement, the question of cultural ownership remains complex. The Cross-Cultural Correction practices identified in the thematic coding suggest that users themselves are engaged in negotiating these questions, collaboratively maintaining cultural accuracy and pushing back against misrepresentations.

The findings also suggest that the lightweight nature of cute aesthetics may limit the depth of cultural understanding that can be achieved through cute IPs alone. While the Muay Thai DunDun Chicken successfully generated affective engagement and cross-cultural interaction, there is little evidence that it led to deeper understanding of Thai or Chinese culture beyond the surface level of cultural symbols. This limitation does not negate the value of cute IPs as tools for transcultural engagement, but it does suggest that they are most effective as entry points rather than as complete vehicles for cultural exchange. Deeper cultural understanding likely requires additional forms of engagement that go beyond the consumption of cute products.

The study’s findings also point to the need for ethical guidelines for the use of cute IPs in cross-cultural contexts. The negative sentiment around cultural symbol misinterpretation suggests that designers and marketers should consult with cultural experts and community members when incorporating cultural symbols into cute IPs. The Cross-Cultural Correction practices identified in the study demonstrate that users themselves can serve as a check on cultural accuracy, but this should not replace proactive efforts to ensure respectful and accurate cultural representation. The balance between cultural depth and lightweight appeal is a delicate one, and achieving it requires ongoing dialogue between designers, marketers, and the cultural communities whose symbols are being represented.

## 6 Conclusion

This study set out to investigate the psychological and cultural dynamics underlying affective engagement with cute IPs across cultures. Our findings provide evidence of what might be termed “transcultural affective resonance”, characterized by similar emotional responses and co-creative participatory practices across Chinese and Thai user groups. However, as we acknowledge in the discussion, our data do not directly demonstrate deeper intercultural understanding or mutual transformation between these groups, which would be the hallmark of a more profound form of transcultural resonance (Rosa, 2019). Rather, the resonance we observed operates primarily at the level of shared affect and participatory engagement. This tension, between the potential of cute IPs to initiate cross-cultural connection and their limitations in fostering deep cultural exchange, represents both a central contribution and a critical boundary condition of our study.

The findings suggest that moe aesthetics function as a broadly shared affective trigger that enables initial emotional engagement across cultural boundaries, consistent with the baby schema framework (Lorenz, 1943; Nittono et al., 2021) and the kama muta response (Steinnes et al., 2019). Both Chinese and Thai users exhibited positive emotional responses to the IP’s visual features, with cute as the most frequent positive keyword on both platforms. However, the interpretive frameworks through which users made sense of their affective experience were culturally situated: Chinese users emphasized product attributes and functional value, reflecting a low-context cultural orientation, while Thai users emphasized cultural identity and symbolic meaning, reflecting a high-context cultural orientation (Hall, 1976). This duality reveals that cute IPs provide a shared affective object around which users from different cultural contexts can gather, while remaining sufficiently open to accommodate different interpretive frameworks.

The thematic coding identified nine participatory practices organized into four thematic clusters: Sensory Breach, Symbolic Hybridity, Participatory Production, and Tribal Fission. These clusters formed a conceptual framework of engagement, from instinctive affective response through creative cultural translation to collective meaning-making and self-organized community governance. The three core categories that emerged from selective coding, Sensory Drive, Symbolic Production, and Tribal Autonomy, constitute the thematic model of transcultural resonance. This model advances a processual understanding of how cute IPs generate transcultural resonance: they begin with universal affective appeal, are transformed through participatory practices into sites of collective meaning-making, and are sustained through self-organized community governance that ensures cultural accuracy while maintaining low barriers to participation.

The cross-cultural comparative analysis revealed that geocultural proximity between China and Thailand significantly facilitated affective engagement, with Thai users exhibiting higher positive sentiment intensity than Chinese users, particularly regarding cultural identity and pride. This finding is consistent with the cultural discount framework (Hoskins and Mirus, 1988). The incorporation of locally meaningful cultural symbols such as Muay Thai ropes and elephant deity imagery reduced cultural barriers and amplified emotional resonance in the Thai context. Yet geocultural proximity alone did not guarantee resonance. Rather, it was the interplay of universal affective triggers, culturally sensitive design, participatory practices, and self-organized community governance that enabled the IP to achieve transcultural resonance.

The study makes several theoretical contributions. First, it extends the concept of transcultural resonance (Klinglmayr, 2024; Rosa, 2019) to the domain of cute IP engagement, offering an empirically grounded framework that specifies the sequential mechanisms through which affective-cultural phenomena generate shared meaning across cultural boundaries. Unlike previous scholarship that has treated resonance as a general orientation toward intercultural communication, the present study operationalizes it through three interconnected layers: Sensory Drive, Symbolic Production, and Tribal Autonomy. Second, the study refines the concept of moe power (Zhao and Niu, 2025) by demonstrating that the affective appeal of cute aesthetics is not uniform across cultural contexts but is actively shaped by local interpretive frameworks and participatory practices. Third, the study contributes to cultural psychology by demonstrating how universal biological predispositions, the baby schema response, interact with culturally specific interpretive frameworks and digitally mediated participatory practices to produce transcultural resonance. This interaction between biological universals and cultural particulars addresses a core concern of cultural psychology: understanding how universal human tendencies manifest in culturally variable ways (Nittono et al., 2021; Lieber-Milo et al., 2025).

The findings also have practical implications for the design and positioning of cute IPs for cross-cultural markets. The negative sentiment identified in the analysis, though comprising only 20.38 percent of comments, clustered around product quality disputes and cultural symbol misinterpretation. The criticism of the elephant deity simplification suggests that designers and marketers should consult with cultural experts and community members when incorporating culturally specific symbols into cute IPs. The Cross-Cultural Correction practices identified in the thematic coding demonstrate that users themselves can serve as a check on cultural accuracy, but this should not replace proactive efforts to ensure respectful and accurate cultural representation. The study also suggests that the lightweight nature of cute aesthetics, while enabling broad affective engagement, may limit the depth of cultural understanding that can be achieved through cute IPs alone. Cute IPs are most effective as entry points for transcultural engagement rather than as complete vehicles for cultural exchange. Deeper cultural understanding likely requires additional forms of engagement that go beyond the consumption of cute products.

Several limitations of this study should be acknowledged. First, the data were drawn from a single IP and two platforms, which limits the generalizability of the findings to other cute IPs, platforms, or cultural contexts. Future research should examine whether the thematic model of transcultural resonance holds for other types of cute IPs, in different cultural pairings, and across other digital platforms. In addition, our removal of emojis during sentiment preprocessing may have reduced our ability to capture affective cues conveyed through visual and contextual markers, such as irony or playfulness, which are common in social media discourse. Future studies could explore multimodal sentiment analysis techniques or employ deep learning models better equipped to handle non-standard text and emojis, thereby providing a more comprehensive picture of digital emotional expression. Second, the sentiment analysis relied on lexicon-based approaches that may not fully capture the nuances of emotional expression in Chinese and Thai social media discourse. Future studies could employ more sophisticated natural language processing techniques or combine computational analysis with in-depth qualitative interviews to capture the full range of emotional and interpretive responses. Third, the study focused on user comments as the primary data source, which captures public expressions of engagement but may not reflect private emotional experiences or the full spectrum of participatory practices. Future research could incorporate surveys, interviews, or ethnographic observation to complement the analysis of user-generated content. Fourth, the cross-cultural comparison was limited to two cultural contexts. Future studies could extend the comparison to include additional cultural groups to examine whether the patterns observed here are specific to the China-Thailand dyad or generalize to other geocultural pairings.

Despite these limitations, the study offers a robust empirical and theoretical foundation for understanding how cute IPs generate transcultural resonance through the interplay of universal affective appeal, culturally situated interpretation, participatory meaning-making, and self-organized community governance. The thematic model of transcultural resonance contributes to cultural psychology by specifying how biological universals and cultural particulars interact in digitally mediated contexts to produce shared affective experiences that transcend cultural boundaries. As cute IPs continue to circulate globally, understanding the psychological and cultural mechanisms that enable or constrain their transcultural resonance becomes increasingly important, not only for commercial success but also for fostering the kind of affective engagement that can contribute to mutual understanding across cultural divides.

## Data Availability

The original contributions presented in the study are included in the article/supplementary material, further inquiries can be directed to the corresponding author.
